# Parent perspectives on guided self-care approaches to ensure timely access to therapy in infants with food allergy

**DOI:** 10.1186/s13223-026-01030-4

**Published:** 2026-04-11

**Authors:** Anna Voia, Marie-Josée Bettez, Catherine Lord, Julie Andrews, Philippe Bégin

**Affiliations:** 1 Allergy Section, Department of Pediatrics, CHU Sainte-Justine, 3175 côte-Sainte-Catherine, Montréal, Canada; 2 Déjouer les allergies, Montreal, QC Canada; 3 Immerscience, Montréal, QC Canada

**Keywords:** Self-care, Food allergy, Oral immunotherapy, Food allergen immunotherapy, Prevention, Healthcare delivery approaches, Qualitative research

## Abstract

**Background:**

Early allergen immunotherapy (AIT), including oral immunotherapy (OIT), is increasingly recognized as a disease-modifying intervention for infants with Immunoglobulin E (IgE)-mediated food allergy. However, limited access to allergy specialists and long wait times often prevent timely initiation during the critical early-life window. Guided self-care models could offer alternative care pathways, yet parents’ perspectives on such approaches remain poorly understood.

**Objective:**

To explore parents’ perceptions of the acceptability, feasibility, and conditions required for guided self-care approaches to to early-life food allergen immunotherapy in infants, drawing on parental experiences managing food allergy in their children in their early years.

**Methods:**

A qualitative study was conducted using semi-structured videoconference interviews (Zoom) with 11 parents of children with food allergies in Québec, Canada, between June and August 2024. Participants were recruited through purposive sampling to capture a range of experiences. Interviews were conducted in French, transcribed verbatim, and analyzed using thematic analysis to identify key themes and constructs in French, translated into English for publication. Rigor was enhanced through independent coding by two analysts, iterative interim analyses, and team discussions to resolve discrepancies.

**Results:**

The mean age of the index child at food allergy diagnosis was 0.8 years (range 0.2–2); four participants were still parents of infants at the time of interview. Three major themes emerged: (1) acceptability of self-care driven by barriers to timely access to AIT and the perceived benefits of early intervention; (2) parental confidence in self-management shaped by experience, preparedness, and education; and (3) the necessity of professional support and clear protocols. Participants expressed strong motivation to initiate early treatment to avoid missing a critical therapeutic window, even in the absence of direct allergist supervision. However, guided self-care was consistently viewed as requiring diagnostic validation, structured protocols, and access to trained healthcare professionals for support, particularly at treatment initiation.

**Conclusion:**

Parents of children with food allergy are receptive to guided self-care approaches for early infant AIT when timely access to specialists is limited. Developing low-risk AIT modalities, standardized protocols, and training for non-allergist healthcare professionals may facilitate earlier intervention and improve outcomes for infants with food allergy.

**Supplementary Information:**

The online version contains supplementary material available at 10.1186/s13223-026-01030-4.

## Introduction

Among the most common chronic conditions in children, Immunoglobulin E (IgE)-mediated food allergies have shown a steady rise in prevalence over recent decades affecting 7.5% of children [1]. Various international guidelines now recommend oral immunotherapy (OIT) as a treatment option to induce desensitization. There is however convincing emerging literature that OIT can be disease-modifying and potentially curative in intent when initiated early in life, before specific IgE have had time to expand [2]. While permanent cure cannot be established with absolute certainty at the individual level, early-life immunomodulatory intervention is increasingly conceptualized as aiming to induce sustained unresponsiveness and long-term remission, thereby altering the natural history of the disease. Various real-life cohorts have confirmed the high safety and supported the effectiveness of early OIT in the infant population [3–5]. In 2025, a randomized control trial showed that a target sublingual dose of only 4 mg protein was effective at inducing remission in infants with peanut allergy, paving the way for alternative, low-intensity forms of allergen immunotherapy (AIT) with very good safety profiles [6].

In light of these new data, early food AIT in infants is now championed as an urgent medical intervention that has the potential to alter the patient’s health trajectory when initiated in a timely manner [7]. Many allergists now prioritize infants and toddlers to discuss and consider AIT before rather than after the window of opportunity to induce tolerance has passed. However, in areas with limited access to specialized care, a shortage of trained allergists and insufficient provider education in food allergy management can result in children waiting years before receiving appropriate evaluation—missing this critical window during which food AIT could have been initiated to promote tolerance through early, supervised exposure.

Limited access to specialized care is far from specific to allergy. Many other specialties have developed alternative care approaches involving other healthcare professionals or self-care programs to improve access to care. Such approaches are less common in the food allergy literature and care. There have been cases of nurse-led clinics to improve access to allergy diagnosis, but not for therapy [8]. AIT is generally considered a highly specialized intervention with several guidelines and reviews specifically mentioning that it should be performed by a specialist with experience at managing anaphylaxis [9, 10]. Recent data showing the high safety of infant OIT, low-dose OIT and sublingual immunotherapy challenge this conception of food AIT as a necessarily high-risk medical intervention. This also contrasts with the general consensus supporting parent-led early introduction of common allergens and the use of food ladders, which may occur outside specialist supervision. While early introduction of most allergens is safe for the majority of children, allergic reactions do occur. Because most allergen introductions take place in children considered at lower risk, a significant number of reactions still occur in this group in absolute terms. This illustrates how parent-led approaches involving small but real risks are already accepted in food allergy care [11–13].

Although introducing self-care elements to early infant food AIT may be technically and clinically feasible, a major knowledge gap remains regarding parents’ willingness to consider alternative forms of care—particularly in the absence of an allergist. This study therefore aimed to explore parents’ perspectives on the possibility and acceptability of self-care approaches in the context of food AIT in infants and conditions under which they would have considered such approaches for their child at the time.

## Methods

Between June and August 2024, we recruited 11 parents to participate in qualitative interviews. Interviews were conducted with only one parent (mother or father). All participants had children with food allergies and most had experience with OIT. The study was approved by the Centre Hospitalier Universitaire Sainte-Justine ethics committee (2024–6505). Participants did not receive compensation or incentives for their participation.

Participants were selected based on their responses to a prior recruitment questionnaire, using a non-probabilistic sampling approach. The purpose of this approach was not to achieve statistical representativeness, but rather to explore a range of experiences and viewpoints in parents with current or past experience with infantile food allergies [14, 15].

Following the electronic endorsement of the consent form via email, participants were invited to partake in a semi-structured individual videoconference interview. All interviews were conducted in French by a citizen-researcher (MJB) trained by a scientific consultant (Immerscience Inc). Participants were provided with the consent form, the list of questions and link to a video explaining OIT in lay terms one week prior to the interview. Consent was reconfirmed verbally at the start of the interviews. Interviews were recorded using Zoom videoconferencing software, de-identified and transcribed in anonymous format for the qualitative analysis. Transcripts were analyzed in French; illustrative quotations were translated into English by bilingual team members and cross-checked to preserve meaning.

The interviewer was supported by a semi-structured interview format, guided by an interview guide co-developed with members of the research team, including a citizen-researcher affiliated with a patient organization (available in supplement). It explored the barriers and facilitators related to early interventions in food allergy—such as OIT and self-care approaches—among parents of children with a history of infantile food allergies. The interview was aimed to last 30 min on average and started with an introductory question on the participant’s experience with food allergy and their general perspectives on AIT, before delving into the topics of (1) early intervention (2) barriers for parents to undertaking AIT to treat their infants’ food allergies, and barriers to infant OIT within our healthcare system and (3) views on administering AIT to an infant at home as parent-delivered care within a guided self-care model, outside of direct specialist supervision, as a form of self-care—assuming there were no medical contraindications— given the shortage of allergists, the delays in accessing specialized care, and the urgency of acting early within the critical treatment window. Open-ended questions were followed by clarification follow-up questions when needed.

### Analysis

Full interview transcripts were analyzed and summarized independently by two team members (AV, JA), who then organized the content thematically to identify key constructs [16]. Partial analyses were conducted after the first three interviews, which allowed for validation of the interview guide. Another interim analysis was performed at the midway point to assess emerging themes. Based on these preliminary analyses and team constant discussion (always excluding the interviewer to avoid contamination of the data), it was determined that data saturation had been reached and decided to conclude data collection after 11 interviews with a diversity of parents with experience with food allergies. The interviewers’ thematic analyses were then consolidated, and any discrepancies were resolved through group discussion involving the interviewer and the four other members of the research team. Participant quotations were selected purposively to exemplify the main themes and subthemes that emerged from the data and to explain participants’ opinions and experiences. They were chosen from various respondents’ perspectives to prevent over-reliance on specific interviews.

## Results

### Description of the sample

A total of 11 interviews were conducted, with an average duration of 34 min (range 21 to 53 min). Table 1 presents the main characteristics of the sample. The participants included 2 fathers and 9 mothers residing in the province of Québec, Canada (see Table 1). All participants had one or more children diagnosed with food allergies. Four participants were parents of infants aged 0–1 years at the time of the interview, with one who had food allergies. The other seven participants were parents of older children who had been diagnosed with food allergies. Three participants had no prior direct experience with OIT.

**Table 1:** Participants characteristics

Participants characteristics	
Mother, n (%)	9 (82)
Father, n (%)	2 (18)
Participant employed in healthcare, n (%)	2 (18)
Family history of allergies, n (%)	6 (55)
Current infant with FA, n (%)	1 (9)
Current infant without FA and older child with FA, n (%)	3 (27)
No current infant with FA, but older child with FA, n (%)	7 (63)
Mean current age (y) of index child (range)	5.9 (0.3–17)
Mean age (y) at first reaction in index child (range)	0.8 (0.2–2)
Multiple food allergies, n (%)	6 (54)
History of anaphylaxis, n (%)	4 (36)
Prior experience with OIT, n (%)	8 (73)
Index child’s FA	
Peanuts	6 (55)
Eggs	6 (55)
Milk	4 (36)
Tree nuts	4 (36)
Seeds	3 (27)
Fish/seafood	2 (18)
Soy, peas, other vegetables	2 (18)

FA, food allergy; OIT, oral immunotherapy

### Thematic analysis results

Thematic analysis of the interviews led to the identification of three major themes: acceptability of self-care; confidence in self-management skills; and support. In this study, the term “self-care” refers to parents undertaking therapeutic actions for their child within a guided care framework, rather than self-management performed by the patient themselves Given the inclusion of both mothers and fathers, we explored whether themes differed by parent gender. No systematic divergences were identified; themes were consistent across participating mothers and fathers.

#### Theme 1 : barriers to timely AIT access and benefits of early interventions drive acceptability to self-care approaches

***The acceptability of guided self-care interventions stemmed from the recognition that lack of timely access to specialized care jeopardized patient’s opportunity to benefit from disease-modifying interventions during their critical time window***. While all participants recognized the allergist’s expertise with regards to OIT and allergy diagnosis, they expressed concerns about timely access to treatment:

“*You have to have a doctor’s prescription for an allergist*,* you have to have the allergist. After that*,* you have to approach OIT itself: can all this really happen within a year?”* [ID#08].

The long delays to see an allergist offering OIT were attributed to the lack of specialists and to the centralization of OIT services in major city centers, Québec and Montréal, Canada. One mother shared her experience of the closure of the only allergy clinic in her region, adding a new geographical barrier [ID#07]. One mother articulated that “*the inadequate channels of communication within the health system*” impeded the accessibility of OIT [ID#09]. Another also mentioned unreliable communications between health services, with “*requests for appointments being misplaced and subsequently mismanaged*” [ID#04].

Participants believed that healthcare professionals lack of knowledge regarding the importance of early intervention could further contribute to delays. One mother emphasized that “*someone who isn’t involved in this wouldn’t know there’s an important window*” for taking action and seeking care [ID#04]. Another participant similarly remarked, “*I don’t understand why such crucial information isn’t even written in* [the free government-issued parenting handbook given to new parents in the province], *which is the Bible*,* the reference everyone uses at the hospital*” [ID#02].

***The perceived benefits of early interventions were to mitigate the risk of severe reactions and enhance patients’ quality of life.*** Participants generally emphasized the preventive aspect of OIT treatment, which could “*perhaps save our children’s lives*” [ID#08], as well as the peace of mind associated with “*less risk*,” which “*took a huge weight off [their] shoulders*” [ID#10]. The impact on daily life was important, both in terms of daily outings and breaking the isolation of their children: “[Before OIT], *we brought our own lunches everywhere we went. The first photo I have of my son eating at a restaurant*,* he was over a year old. We had never dared to before. That’s a big deal*.” [ID#10]. As most participants with prior experience stated, OIT has “*completely changed [their] life*” [ID#03].

*All participants felt the long-term advantages outweighed the risks of early intervention.* Although participants acknowledged the difficulties related to OIT, including the risk of allergic reactions, disruptions to daily routines, and the significant time commitment required for appointments, every participant felt that the long-term advantages outweighed the risks, they described these inconvenient as relatively minor—sometimes even “*minimal*”—when compared to the dangers of not pursuing OIT [ID#01]. One mother said she was “*more focused on the future benefits*,* despite possible effects*” in the short term [ID#07], while another noted that any reactions, though unsettling, “*were not a danger to* [her daughter’s] *immediate physical health*” [ID#09].

#### Theme 2: parents’ confidence in self-management shaped by experience and preparedness

***Inadequate self-management of allergic reactions was identified as the participants’ main concern, regardless of the treatment method, self-care or not***. When asked to elaborate on their fear of mismanaging reactions in the context of a self-care intervention, all participants spontaneously pivoted to reflecting on a previous experience with medically guided OIT or accidental exposures. Participants described the feeling of inadequate competence when managing allergic reactions. Insecurities about recognizing the signs of an allergic reaction and using the [epinephrine auto-injector] were described as generating “*quite a bit of stress*” [ID#05], both inside and outside the context of OIT. Several participants stressed the importance of parental preparedness: “T*o reduce anxiety*,* it requires education*” [ID#10].

One mother highlighted the challenge of recognizing symptoms in an infant: “*He’s crying*,* but what’s going on? Is this a reaction that’s taking place?*” [ID#08]. Another worried that symptoms could go unnoticed while they develop slowly or remain hidden: *Sometimes you can’t see it clearly until you take a bath or something*,* and then the color shows up*” [ID#02].

***Experiential knowledge contributes to confidence in self-management skills***. Participants with a family history of food allergies thought it contributed to a higher level of knowledge and comfort with the OIT process. In reflecting on the experience with her first child, one participant shared that undergoing OIT at the time would have been “*too overwhelming*,* too intense*,* too risky even […] The mom I was then wouldn’t have been able to handle it*” [ID#03]. Participants believed that parents of a first child could be more hesitant to expose their baby to potentially dangerous allergens: “*Having a first baby already requires support*,* and now you also have to expose them to potentially dangerous foods*” [ID#11]. The mother of an infant and two older children with food allergies explained that starting OIT with a second child would be easier: *“In our particular case*,* I think I’d be comfortable because we know the symptoms*,* and we know how to respond because of our history*” [ID#11].

***Lack of personal experience can be overcome through access to proper training and information***. Even assuming they had no prior experience with allergic children, most participants expressed a willingness to undertake a form of self-care intervention involving food ingestion at home, provided they were adequately informed about the intervention. One participant shared: “*I would still do it if I had the necessary information*” [ID#08]. One father noted, “*Give us 2 hours of content*,* explain what it is*,* what the risks are. It’s much easier when we have a video of someone explaining*,* rather than reading*” [ID#02].

***Participants felt they had low competency to self-diagnose food allergy***. Participants identified confirming diagnosis as a prerequisite before engaging in self-care. One participant explained “*the diagnosis must be made before parents can decide to continue at home*” [ID#08]. One participant mentioned that: “*The diagnosis must be made by an allergist; there’s no way around it. My point of view is that it takes expertise*,* it takes time*” [ID#02]. Others suggested the use of skin tests by other professionals, such as nurses or family doctors, to reduce diagnosis delay. *“It’s not all allergists that do IgE. Most of them just do skin test. […] It seems easy enough to do. I’m pretty sure other professionals could do it.”* [ID#04].

The option of self-diagnosing food allergy with a questionnaire was also raised. As one mother put it: “*Personally*,* I have this skill*,* I think*,* that for my [third] little boy*,* I would have felt comfortable doing a questionnaire and then determining it myself. With* [my second], *I would have known*,* but I don’t think I had all the tools yet*,* so I might have needed a little more guidance there*” [ID#11]. Participants raised concerns about the risk of missing an allergy or falsely identifying a non-existent one. However, one mother pointed out that this is not a new issue, since parents already face the dilemma when deciding whether to remove foods from her child’s diet based on a possible allergic reaction: “*Maybe it wasn’t an allergic reaction; I’m confused… but I’m going to cause him an allergy he didn’t have* [if I remove the food]” [ID#05].

#### Theme 3: effective self-care depends on valid professional support and clear protocols

***Self-care does not mean doing it alone***. While all participants were open to the possibility of self-care, their conception of self-care always included the participation of a healthcare professional to some degree. The perceived requirement for support by a healthcare professional varied according to stage of treatment. For example, all participants felt an initial contact with a healthcare professional was necessary at least to confirm diagnosis and eligibility, the absence of contraindication to OIT or self-care and possibly for a supervised first introduction of the food. Professional supervision answered a need for validation: “*It can be reassuring if someone with medical training gives the stamp of approval*” [ID#11]. After initiation of treatment however, most participants thought they probably would not require direct support. “*The rest would be done independently*,” explained one mother [ID#10]. However, some participants felt that every new dose should be validated by a healthcare professional.

***Self-care requires clear and precise protocols and a point of contact with healthcare for emerging issues***. Most participants expressed the need for clear guidelines to *“feel equipped enough to know the quantity*,* the frequency*,* to really have the right protocol to follow”* [ID#09]. *“It has to be really clear in advance: at such and such week*,* we introduce such and such food at such and such dose*” [ID#11]. Very low starting doses were identified as a potential problem: *“It was so microscopic*,* the dose was like a grain of sand”*, explained one paticipant [ID#10].

Participants emphasized the value of having an open line of communication with a trusted professional, especially at the beginning when they are still developing their self-management skills. One father explained that “*there’s an open line—if anything happens*,* we can call that person. Then you feel there’s support and*,* like with everything*,* that builds trust*” [ID#01].

Tools mentioned by participants as potentially useful included a support line, teaching material with videos, access to peer support, phone applications and symptom diaries.

***Involvement of non-allergist healthcare professionals is conditional on them having received relevant training***. Participants mentioned family doctors and nurses as potential alternatives to allergists in overseeing food allergy guided self-care interventions. Pharmacists were seen as a reliable source of information regarding management of reactions and understanding the medication involved. One mother with previous experience with OIT mentioned a role for nutritionists. “*She guided us a lot because she knows about substitute products*” [ID#10]. One participant summarized that support could be provided by “*any professional who can manage an allergic reaction*,* give the Epipen*,* stay calm*,* and monitor vital signs*” [ID#06]. One mother emphasized that the professional needs to know “*how to take the lead if something happens*,* and who helps you think clearly when you’re under stress*” [ID#03].

One mother expressed concerns that her current family doctor “*lacked the tools to answer [her]*” when it came to food reintroduction, often feeling more informed on the topic herself [ID#10]. Another participant highlighted inconsistencies in the advice she received: “*The information provided by my family doctor was not aligned with that of the allergist*” [ID#09]. Participants mentioned such discrepancies could have a detrimental effect on the patients’ level of trust in the treatment process, highlighting the need to inform healthcare professionals beyond only those who would take active part in an eventual self-care program.

## Discussion

To our knowledge, this is the first study exploring parents’ perspectives on alternative care pathways to allow timely delivery of food allergy interventions in infants with food allergy. Several studies have explored parental perspectives on oral immunotherapy more broadly, but little work has examined parental views on alternative care pathways aimed specifically at enabling early intervention in infants [17]. Despite the risk of reactions, participants expressed their willingness to initiate treatment to their infant through guided self-care involving healthcare professionals, in order not to miss the window of opportunity to induce remission.

A key finding was that participants’ conception of self-care systematically involved at least some interaction with a health care provider. In the literature, this is referred to as guided self-care, by opposition to self-directed self-care. In a review of self-care clinical trials in psychiatry, McCusker et al. (2023) found that while self-directed approaches significantly improved patient outcomes, guided self-care was more effective in the treatment of depression and resulted in greater user engagement. The effectiveness of guided self-care interventions depended on factors such as the number of support calls and the presence of additional interventions, and the complexity of delivery [18]. This echoes our participants’ comments regarding the need for support, which was found to vary depending on treatment stage, with diagnosis being identified as the most complex element for patients to navigate and for which professional support would be most required. Our participants further identified previous experience with a child with food allergy or with food allergy in general as a facilitator for self-guided interventions. In their opinion, it was not however an absolute requirement and they suggested that lack of experiential knowledge could be compensated by proper education and guidance. Participants also underscored the high level of intrinsic motivation in this population, once provided with the right information, which is another key factor of success in self-care literature [19].

In the food allergy literature, the concept of self-care usually refers to self-management of accidental reactions [20, 21]. This said, while it may not be perceived as such, there are other examples of interventional self-care approaches in food allergy. One would be home-based OIT protocols where patients increase doses at home. These protocols usually involve patients that have been carefully evaluated by an allergist and does not directly solve the issue of difficult access to a specialist [4, 22]. A more apt comparator could be food ladders, which are widely recommended by allergists and non-allergists alike. While they are not without risk, their use is similarly justified by the lack of access to specialist or food challenge platforms [11]. The purest example of self-care in food allergy is probably the recommendations for early introductions of allergens in high-risk children without prior testing, in which allergists fully embrace a self-directed self-care approach and where a certain risk of reaction is justified from a public health standpoint [12].

The literature identifies multiple determinants that contribute to patient’s tolerance to risk in the context of self-care (Table 2), all of which were spontaneously mentioned by our participants to various extents [23–25]. One key determinant of tolerance to self-care is condition stability. While food allergy is generally viewed as a chronic, relatively stable condition, the intervention itself can introduce significant instability, leading to unforeseen acute or even urgent care situations. Accordingly, our participants’ discourse on risks and concerns naturally centered on the specific context of up-dosing or co-factor-induced reactions at higher dosages. The acceptability of self-care AIT interventions may hinge on the removal of these barriers. Approaches that avoid up-dosing such as sublingual, epicutaneous, or very low-dose oral immunotherapy, would likely be more amenable to guided self-care than a traditional OIT protocol pushing for maintenance doses above the patients baseline reactivity threshold. This said, even with lower-risk AIT alternatives, the need for diagnostic validation by a healthcare professional precludes a fully self-directed approach. 

**Table 2:** Determinants of Risk tolerance in guided self-care (parent-delivered care)

Individual factors
Confidence, health literacy, psychological traits, motivation, past experiences.
Clinical factors
Severity of condition, stability, physical ability to perform self-care.
Social factors
Support, cultural norms and expectations.
System factors
Access to healthcare, availability of tools, telehealth, affordability.
Intervention factors
Complexity of required self-care and its potential consequences.

Practical implications of our findings include the need to further explore AIT interventions that do not require up-dosing and to train more healthcare professionals beyond only allergy specialists, on not only their delivery, but also on the timely recognition and diagnosis of food allergy in infants. This is particularly important as misdiagnosis or inappropriate testing by insufficiently trained providers can lead to unnecessary dietary avoidance and additional stress for families. It also points to the need to develop clear and validated protocols that will be accessible and available in various adapted formats for healthcare professionals and for parents.

This study had some limitations. Most participants were motivated individuals from a food allergy patient community, which may contribute to some polarization of reported opinions (volunteer bias). Also, many of the participants were aware of the research team’s previous work and involvement in new food allergy intervention, which could have affected the way they responded (desirability bias). It is thus important to remember that the primary objective of a qualitative study is not to generalize results, but rather to explore and understand perspectives on the issue. Finally, none of the participants had actually experienced an early self-care intervention for their infant. Those with experience in OIT had done so with older children and under medical supervision. Transferability considerations include that most participants were recruited from a highly engaged food allergy community in Québec, and many had prior OIT experience. In addition, although the study focus was early-life intervention, many participants’ index children were school-aged at the time of interview, meaning some perspectives were retrospective. These contextual factors should be considered when applying findings to other healthcare systems or less experienced parent populations.

## Conclusion

In conclusion, our study found that allergy-experienced parents are in favor of developing guided self-care approaches in infants with a recent reaction to a food allergen to allow early initiation of time-sensitive interventions. The use of AIT approaches that do not require up-dosing could remove a significant source of risk and barriers that were identified by participants. Clinical guidance was still deemed required to confirm diagnosis, validate treatment plan and provide support in case of reactions. To their view, this guidance could be provided by allied healthcare professionals, contingent on adequate training and standardized protocols for both health care teams and parents. This study paves the way for initiatives exploring a shift toward innovative self-care approaches that could reduce treatment delays and improve outcomes for infants with food allergy.

## Supplementary Information

Below is the link to the electronic supplementary material.

Supplementary Material 1

## Data Availability

The datasets generated and analyzed during the current study are not publicly available due to the qualitative nature of the data and the risk of participant identification, but are available from the corresponding author on reasonable request.

## Abbreviations

AIT: Allergen immunotherapy
IgE: Immunoglobulin E
OIT: Oral immunotherapy
