# Supplementary material for: Parent perspectives on guided self-care approaches to ensure timely access to therapy in infants with food allergy
Source: Allergy Asthma Clin Immunol. 2026 Apr 11;22:34. doi: 10.1186/s13223-026-01030-4 (PMC13214098; doi:10.1186/s13223-026-01030-4)
Supplement: Supplementary file 1 — Supplementary Material 1 [file 13223_2026_1030_MOESM1_ESM.pdf]

GUIDE D'ENTRETIEN SEMI-DIRIGÉ - RECHERCHE PARTICIPATIVE SUR L'ACCÈS À L'IMMUNOTHÉRAPIE ORALE (ITO) PRÉCOCE CHEZ LES NOURRISSONS

Logistique (générale)

Les participants potentiels seront contactés par courriel ou téléphone. Après relecture et échange des signatures électroniques du consentement par courriel, s'ils sont toujours intéressés, ils seront invités à participer à un entretien individuel semi-structuré sur Zoom.

Une semaine avant l'entretien, les participants recevront la liste des questions ainsi que le lien vers une capsule avec Dr Philippe Bégin sur l'ITO et son fonctionnement. L'objectif est de leur permettre de réfléchir d'avance à leurs réponses et d'organiser leur pensée.

Les entretiens seront enregistrés localement sur l'ordinateur portable de l'intervieweuse. Les enregistrements seront ensuite transférés sur le serveur sécurisé du centre de recherche du CHU Sainte-Justine. Chaque entretien sera transcrit et dénominalisé pour être analysé. Les entretiens seront supprimés de l'ordinateur de l'intervieweuse après leur transcription.

Les données seront conservées pendant sept ans sur le serveur du centre de recherche du CHU Sainte-Justine.

Étapes et scripts

1. **Envoi d'un courriel pour fixer la date du pré-entretien téléphonique**

   Objet : Votre participation à un projet de recherche sur l'immunothérapie orale chez les nourrissons

   Cher monsieur X,

   Chère madame Y,

   Tout d'abord, permettez-moi de vous remercier d'avoir complété, en août dernier, le sondage de Déjouer les allergies sur l'immunothérapie orale (ITO) précoce chez les nourrissons. À la fin de ce sondage, vous avez exprimé votre intérêt à participer à notre étude menée en collaboration avec le Dr Philippe Bégin, allergologue au CHU Sainte-Justine. C'est pour savoir si vous êtes toujours intéressé à participer à ce projet que je vous écris aujourd'hui.

   Je précise qu'il n'y aura pas de traitement d'ITO dans le cadre de cette étude. Celle-ci s'adresse en effet exclusivement aux parents et repose sur un entretien via Zoom afin de mieux comprendre leur point de vue sur le sujet de l'ITO chez les nourrissons.

   Concrètement, votre participation se déroulera en deux temps :

- *un court appel téléphonique pour s’assurer que les critères de sélection soient remplis et obtenir votre consentement*
- *le cas échéant, un entretien sur Zoom d’une durée maximale de 45 minutes pour discuter de l'ITO chez les nourrissons*

Je serai la seule membre de l'équipe de recherche à vous parler lors de l'appel téléphonique et de l'entretien sur Zoom. Vos données seront anonymisées et utilisées uniquement dans le cadre de cette étude.

Seriez-vous disponible, au cours des prochains jours, pour l’appel téléphonique initial ? Si oui, quel serait le meilleur moment pour que je vous appelle ?

Je vous remercie à l’avance pour votre réponse et pour votre engagement envers ce projet de recherche important.

Cordialement,

2. Appel téléphonique

Bonjour,

Je m’appelle Marie-Josée Bettez et je suis la directrice générale de l’organisme Déjouer les allergies.

Tout d’abord, merci pour votre intérêt à l’égard de notre étude sur l’accès à l’ITO précoce chez les nourrissons.

Comme je vous l’ai mentionné dans mon courriel, nous menons cette étude en collaboration avec Dr Philippe Bégin, allergologue au CHU Sainte-Justine. L’objectif de ce projet est d’explorer les pistes de solution permettant aux familles d’initier une ITO chez les nourrissons, avant même d’avoir rencontré l’allergologue.

Je vous appelle aujourd’hui pour m’assurer que vous remplissez les critères de sélection de l’étude et, si c’est le cas, pour obtenir votre consentement à y participer.

Si cela vous convient, j’ai deux ou trois questions à vous poser :

- *Êtes-vous le père/mère d’un enfant ayant une ou plusieurs allergies alimentaires?*
- *Si oui : quel est l’âge de cet/ces enfant.s? Combien d’enfants avec et sans allergies?*

Si le participant est le parent d’un enfant allergique ou le parent d’un enfant de moins d’un an sans histoire d’allergies alimentaires :

Vous répondez effectivement aux critères de sélection.

Avant d’aller plus loin, avez-vous des questions ou des commentaires sur le projet de recherche ? Êtes-vous toujours intéressé à y participer?

Je vais vous transmettre par courriel un document de 4 pages contenant des informations sur la recherche et incluant le formulaire de consentement à signer. Je vais maintenant vous lire la section sur le consentement (vous pourrez lire le reste du document de votre côté) :

***«** On m'a expliqué la nature et le déroulement du projet de recherche. J'ai pris connaissance du formulaire de consentement et on m'en a remis un exemplaire. J'ai eu l'occasion de poser des questions auxquelles on a répondu. Après réflexion, j'accepte de participer à ce projet de recherche.*

En signant ce formulaire de consentement, vous ne renoncez à aucun de vos droits prévus par la loi. De plus, vous ne libérez pas les investigateurs de leur responsabilité légale et professionnelle advenant une situation qui vous causerait préjudice.

Je vous ai expliqué tous les aspects pertinents de la recherche et j'ai répondu à vos questions. Je vous ai indiqué que la participation au projet de recherche est libre et volontaire et que la participation peut être cessée en tout temps. »

Une fois le formulaire signé, je vous proposerai par écrit des dates pour un appel Zoom d'une durée maximale de 45 minutes. Y a-t-il des journées et des moments dans la journée qui sont préférables pour vous ?

Merci de m'avoir consacré ces quelques minutes et merci encore pour votre intérêt à l'égard de notre projet de recherche.

À bientôt !

Transmettre le formulaire de consentement par courriel.

3. Courriel pour obtenir le consentement écrit

Objet : Formulaire de consentement

Cher monsieur X,
Chère madame Y,

Pour faire suite à notre récente conversation téléphonique, vous trouverez ci-joint un document explicatif sur le projet de recherche « Élargir l'accès à l'immunothérapie orale précoce chez les nourrissons » ainsi qu'un formulaire de consentement. Je vous invite à lire ce document et, si vous acceptez toujours de participer à cette étude, à le signer. J'apprécierais si vous pouviez me retourner le tout par courriel d'ici le JJ/MM/2024.

Comme je vous l'ai déjà mentionné, votre participation est libre et volontaire et peut être cessée en tout temps.

Je vous remercie à l'avance pour votre collaboration et votre soutien à cette recherche.

Cordialement,

Joindre à ce courriel le document « Information sur la recherche et formulaire de consentement »

Sur réception du consentement signé, contresigner celui-ci.

4. Courriel pour fixer la date de l'entretien sur Zoom

Objet : Date de l'entretien sur Zoom

Cher monsieur X,
Chère madame Y,

Je vous remercie d'avoir signé le formulaire confirmant que vous consentez à participer au projet de recherche « Élargir l'accès à l'immunothérapie orale précoce chez les nourrissons ». Vous trouverez ci-joint une copie contresignée de celui-ci.

Voici quelques propositions de dates pour l'entretien de 45 minutes sur Zoom :

- *JJ/MM/2024, heure*
- *JJ/MM/2024, heure*
- *JJ/MM/2024, heure*

Merci de m'indiquer dès que possible celle qui vous convient le mieux.

Cordialement,

Joindre le consentement contresigné.

5. Envoi des questions (une semaine avant l'entretien sur Zoom)

Objet : Lien Zoom et questions

Cher monsieur X,
Chère madame Y,

Un mot pour vous rappeler que notre entretien sur Zoom dans le cadre du projet de recherche « Élargir l'accès à l'immunothérapie orale (ITO) précoce chez les nourrissons » se déroulera le JJ/MM/2024, heure. Le lien pour vous connecter est le suivant : XXX

Pour vous permettre de vous préparer à cette rencontre, voici la liste des questions qui seront posées :

- *Pouvez-vous me parler de votre expérience personnelle en lien avec les allergies alimentaires ?*
- *Pouvez-vous me parler de votre expérience personnelle en lien avec l'ITO ?*

- *Quelles sont, selon vous, les barrières pour les parents à faire de l'ITO pour traiter les allergies alimentaires de leurs nourrissons ?*
- *Quelles sont, selon vous, les barrières à l'ITO chez les nourrissons dans notre système de santé ?*
- *On sait que l'ITO est plus efficace lorsqu'elle est réalisée au cours de la première année de vie. On sait aussi qu'il n'y a pas suffisamment d'allergologues au Québec pour initier le traitement rapidement chez tous les nourrissons allergiques. Dans ce contexte, en supposant qu'il n'y ait pas de contre-indications à l'ITO, sur le plan médical, pour un nourrisson particulier, le vôtre par exemple, que penseriez-vous de faire de l'ITO en auto-soin à la maison pour traiter ce nourrisson ?*
- *Est-ce qu'il y a d'autres problèmes en lien avec l'ITO chez les nourrissons que nous n'avons pas abordés et qui vous semblent préoccupants ?*

Pour en savoir plus sur l'ITO et son fonctionnement, je vous invite à visionner cette courte capsule avec Dr Philippe Bégin avant notre rencontre : https://youtu.be/MlA5Ryrw37U?si=p23N-m2IcMhlED2Q

Je vous remercie à l'avance pour votre collaboration et votre soutien à cette recherche.

Cordialement,

6. Entretien sur Zoom

Toujours garder en tête que la grille d'entretien doit être utilisée de manière souple. Elle peut être adaptée au besoin, selon les questions auxquelles on cherche à répondre, qui est interviewé.e et le niveau de connaissances sur les allergies alimentaires et/ou l'ITO de l'informateur.trice (parents ou professionnel.les de la santé).

Par exemple, vous pouvez répondre aux questions des informateur.trice.s selon votre niveau de connaissance en prenant soin de ne pas influencer leurs opinions, mais en rectifiant les faits, si et quand vous le jugerez opportun. Il peut être judicieux de revenir sur certains points à la fin de l'entrevue, par exemple pour pousser un peu plus loin et ce, sans interférer avec la collecte du propos originel de l'informateur.trice.

La première section de la grille proposée porte sur la présentation de l'intervieweur.euse . C'est un exemple à adapter. L'important est de se présenter et de redonner les thèmes qui seront abordés pendant l'entretien (les thèmes ayant déjà été présentés lors du recrutement et de la signature du formulaire de consentement, entre autres). Ainsi, l'informateur.trice sait exactement quelles sont les attentes de l'intervieweur.euse avant que la collecte de données commence.

La deuxième section constitue le cœur de la grille d'entretien. Elle rappelle les thèmes à discuter et suggère des formulations de questions. La grille d'entretien propose deux séries de questions, dont l'une porte sur le thème des problèmes de l'ITO et l'autre sur l'appréciation des services à la population.

Des exemples de formulation de questions y sont proposés. Le but n'est pas de poser toutes les questions comme on le ferait dans le cas d'un questionnaire fermé, par exemple, mais de suivre la trame générale.

Dans le cas des problèmes d'ITO, il est important :

- d'obtenir des renseignements sur les problèmes d'ITO vécus par la personne ; et
- d'amener l'informateur.trice à définir les problèmes qu'il.elle juge les plus préoccupants.

Pour ce qui est des questions sur la faisabilité de l'ITO à la maison, il est important de maintenir le cap sur le fait que l'ITO à domicile ne se fait pas s'il y a des contre-indications chez le nourrisson.

Afin de faciliter l'enchaînement et de vous assurer d'avoir bien saisi le propos de l'informateur.trice, il peut être utile de résumer le thème discuté précédemment avant de passer à un autre sujet. Vous pouvez redire dans vos mots ce que vous avez retenu et laisser l'informateur.trice rebondir et préciser ses propos, au besoin.

À chaque changement de thème, le recours à des questions principales, plus générales, facilite le déroulement. Dans tous les cas, l'intervieweur.euse doit en choisir une avec laquelle il est à l'aise et qui est adaptée au niveau de connaissance et à la position face aux allergies et à l'ITO chez les nourrissons de l'interlocuteur.trice. La question sera formulée différemment, par exemple, si elle s'adresse à un.e citoyen.ne qui mentionne être un.e débutant.e en matière d'ITO.

Dans l'exemple présenté, des questions de clarification sont suggérées. Le recours à ce type de questions permet d'obtenir des précisions. Avoir en tête quelques versions de ces questions est nécessaire afin de susciter la discussion et assurer la fluidité des échanges.

Si l'informateur.trice a de la difficulté à nommer des problèmes d'ITO, l'intervieweur.euse peut lui donner des exemples de thèmes, sans toutefois lui suggérer des réponses.

La troisième partie vise à bien clôturer l'entretien. Pour ce faire, deux questions sont suggérées.

Avant de terminer l'entretien, il faut s'assurer que les thèmes ont été couverts le plus possible. Prenez quelques minutes pour revoir vos notes et revenir au besoin sur certaines « zones grises » qui auraient émergé pendant l'entretien.

En tout dernier lieu, il est important de remercier votre informateur.trice pour sa participation essentielle à cette recherche et de prendre le temps de lui expliquer la suite du projet de recherche et de répondre à ses questions en lien avec l'entretien, le présent projet ou la recherche scientifique.

Grille d'entretien

**Présentation**	
Intervieweuse	*Bonjour, merci d'avoir accepté de participer à ce projet de recherche sur l'accès à l'ITO précoce chez les nourrissons.*<br><br>*Je m'appelle Marie-Josée Bettez. Je suis la directrice générale de l'organisme Déjouer les allergies. C'est moi qui vous interviewerai aujourd'hui mais c'est l'équipe de recherche au complet qui analysera vos réponses à partir de l'enregistrement de cette entrevue.*<br><br>***Si vous n'y voyez pas d'inconvénient, je vais lancer l'enregistrement maintenant** (à partir de ce moment, ne plus référer au participant par son nom)*<br><br>*Vous avez déjà reçu le formulaire d'information et de consentement et vous avez signé celui-ci. Pouvez-vous me confirmer verbalement que vous acceptez toujours de participer au projet de recherche ?*<br><br>*Nous pouvons maintenant débuter formellement l'entrevue.*<br><br>*Je fais équipe avec Dr Philippe Bégin du CHU Sainte-Justine pour mettre en lumière les freins et les facilitateurs à l'accès à l'ITO chez les nourrissons.*<br><br>*Au cours de l'entretien, j'aimerais que nous abordions les thèmes suivants :*<br><br>*1- votre expérience personnelle en lien avec les allergies alimentaires et l'ITO*<br>*2- vos opinions en lien avec l'ITO chez les nourrissons*<br><br>*En ayant ces thèmes en tête, … (début de l'entrevue)*

<table>
<tr><th>Thématiques</th><th>Sous-questions</th><th>Exemples</th><th>Notes</th></tr>
<tr><td rowspan="3">Pouvez-vous me parler de votre expérience personnelle en lien avec les allergies alimentaires ?
ou
Avez-vous un ou des enfants qui ont des allergies alimentaires ?</td><td></td><td></td><td rowspan="3">Il s’agit de la première question pour tous les entretiens.</td></tr>
<tr><td></td><td></td></tr>
<tr><td></td><td></td></tr>
<tr><td>Pouvez-vous me parler de votre expérience personnelle en lien avec l’ITO ?
ou
Avez-vous un ou des enfants qui ont suivi un traitement d’ITO ?</td><td>Durée, résultat, difficultés, impact sur la qualité de vie…
Autre chose à ajouter sur votre expérience personnelle en lien avec ITO ?</td><td></td><td></td></tr>
</table>

Quelles sont, selon vous, les barrières pour les parents à faire de l'ITO pour traiter les allergies alimentaires de leurs nourrissons ?<br><br>*ou*<br><br>*Selon votre expérience personnelle ou professionnelle, quels sont les freins pour les parents à faire de l'ITO pour traiter les allergies alimentaires de leurs nourrissons ?*<br><br>*ou*<br><br>*Selon vous, qu'est-ce qui pourrait empêcher les parents de faire de l'ITO pour traiter les allergies alimentaires de leurs nourrissons ?*	*Comment percevez-vous le risque associé à ce traitement ?*	*Accès aux ressources*<br><br>*Manque de temps*<br><br>*Incapacité des nourrissons à s'exprimer*<br><br>*Manque de support*	

Quelles sont, selon vous, les barrières à l'ITO chez les nourrissons dans notre système de santé ?<br><br>*ou*<br><br>*Selon votre expérience personnelle ou professionnelle quels sont les freins à l'ITO chez les nourrissons dans notre système de santé ?*	*Est-ce que vous pensez que des professionnel.le.s de la santé autre que les allergologues pourraient superviser l'ITO à la maison?*	*Infirmières, médecins de famille, pédiatres, etc.*	
	Est-ce que vous pensez que des professionnel.le.s sans formation médicale pourraient superviser l'ITO à la maison?	*Nutritionnistes, etc.*	

On sait que l'ITO est plus efficace lorsqu'elle est réalisée au cours de la première année de vie. On sait aussi qu'il n'y a pas suffisamment d'allergologues au Québec pour initier le traitement rapidement chez tous les nourrissons allergiques.<br><br>*Dans ce contexte, en supposant qu'il n'y ait pas de contre-indications à l'ITO, sur le plan médical, pour un nourrisson particulier, le vôtre par exemple :*<br><br>*Que penseriez-vous de faire de l'ITO en auto-soin à la maison pour traiter ce nourrisson?*<br><br>*ou*<br><br>*Afin de pallier aux délais d'accès et agir tôt, que pensez-vous de faire de l'ITO en auto-soin pour traiter ce nourrisson dans l'attente d'une prise en charge par un.e professionnel.le de la santé?*	*Selon vous, qu'est-ce que l'auto-soin?*		*Exemple d'auto-soin : vitamines prénatales (acide folique) pour les femmes enceintes.*
	Comment est-ce que l'absence de contre-indications à l'ITO chez un nourrisson en particulier devrait être établie?	*Auto-diagnostic sur la base d'un questionnaire*<br><br>*Diagnostic d'un.e professionnel.le de la santé*	
	Selon vous, dans quelles conditions serait-il acceptable que l'ITO soit réalisée entièrement à la maison par les parents de ce nourrisson ?		
	De quel genre de support auraient besoin les parents de ce nourrisson pour faire l'ITO à la maison?		

	Afin de gérer le stress et la peur d'une réaction allergique déclenchée par l'ITO à la maison, de quel genre de support aurait besoin les parents de ce nourrisson?		

<table>
<tr><th colspan="4">Clôture</th></tr>
<tr><td rowspan="3">Est-ce qu'il y a d'autres problèmes en lien avec l'ITO chez les nourrissons que nous n'avons pas abordés et qui vous semblent préoccupants ?
ou
Avez-vous quelque chose d'autre à ajouter concernant l'ITO chez les nourrissons ?</td><td></td><td></td><td rowspan="3"></td></tr>
<tr><td>Vous avez mentionné ceci en début d'entrevue, est-ce que je résume bien vos propos : … ?</td><td></td></tr>
<tr><td>Avez-vous d'autres questions sur l'entretien ou le projet de recherche ?</td><td></td></tr>
<tr><td colspan="4">Cela conclut cette entrevue.
Merci beaucoup d'avoir participé à cette recherche. Au cours des prochaines semaines, nous poursuivrons les entrevues avec les participants puis nous les retranscrirons intégralement pour les analyser.
Les résultats de l'étude seront d'abord présentés à la communauté Déjouer les allergies puis ils seront diffusés dans des publications scientifiques et feront l'objet de présentations à diverses instances.
Avant de se laisser, avez-vous d'autres questions à propos de cette entrevue ou du projet de recherche ?</td></tr>
</table>

Notes générales de l'intervieweuse :
